# Investigation of single and multiple MPPT structures of solar PV-system under partial shading conditions considering direct duty-cycle controller

**DOI:** 10.1038/s41598-023-46165-1

**Published:** 2023-11-03

**Authors:** Abdel-Raheem Youssef, Mostafa M. Hefny, Ahmed Ismail M. Ali

**Affiliations:** Electrical Engineering Department, South Valley University, Qena, 83523 Egypt

**Keywords:** Energy science and technology, Mathematics and computing, Engineering, Electrical and electronic engineering

## Abstract

Partial shading of solar panels diminishes their operating efficiency and energy synthesized as it disrupts the uniform absorption of sunlight. To tackle the issue of partial shading in photovoltaic (PV) systems, this article puts forward a comprehensive control strategy that takes into account a range of contributing factors. The proposed control approach is based on using multi-string PV system configuration in place of a central-type PV inverter for all PV modules with a single DC-DC converter. This adaptation enhances overall efficiency across varying radiation levels. Also, the proposed technique minimizes the overall system cost by reducing the required sensors number by utilizing a radiation estimation strategy. The converter switching strategy is synthesized considering direct duty-cycle control method to establish the maximum power point (MPP) location on the P–V curve. The direct duty-cycle tracking approach simplifies the control system and improves the system’s response during sudden partial shading restrictions. To validate the effectiveness of the suggested MPPT method, two system configurations were constructed using MATLAB/SIMULINK software and assessed under various partial shading scenarios. Additionally, a multi-string system was subjected to real irradiance conditions. The sensor-less MPPT algorithm proposed achieved an impressive system efficiency of 99.81% with a peak-to-peak ripple voltage of 1.3V. This solution offers clear advantages over alternative approaches by reducing tracking time and enhancing system efficiency. The system findings undoubtedly support the theoretical scrutiny of the intended technique.

## Introduction

Renewable energy sources (RESs) are gaining prominence worldwide as a response to urgent environmental challenges caused by fossil fuels. For instance, global warming, the nature destruction, and water contamination that lead to worst climate impacts^1–3^. Solar photovoltaic (PV) systems have emerged as a feasible answer to address the increasing global electricity demands. The combined installed capacity of the solar PV market stands at 892.6 GW and is projected to experience a compound annual growth rate (CAGR) exceeding 15% from 2021 to 2030^4^. The enhancement of solar energy efficiency has gained a significant alertness from scholars, owing to its environmentally friendly nature, consumer expenses reduction, and encourage economic expansion^5^. However, PV-based generation systems undergo numerous encounters, involving excessive installation expenditures, low efficiency, and climate reliance^6,7^. The stochastic nature of the solar PV system under continuous varying weather conditions leads to voltage\frequency fluctuation at the coupling point with the utility network, which leads to an intermittent and unstable consumer power service^8^. Many factors influence the operation of the solar PV system. One of the most familiar issues related to weather dependence is partial shading condition due to existence of clouds, dust on the surface of the panel, etc. The performance of the entire PV system is negatively impacted by even light shade that may leads to PV system destruction^9,10^. In such conditions, Shaded PV modules generate lower electrical current compared to the unshaded surroundings, and a larger passing current that would heat them up and causing a hot-spot problem that could harm those arrays^11^. This issue can be resolved by connecting a bypass diode in shunt, which will cause the current to go through a forward-biased diode^12,13^. In fact, the existence of partial shading condition (PSC) results in multiple local maximum power points (LMPPs) and a unique global maximum power point (GMPP). Therefore, many maximum power point tracking (MPPT) techniques have been recommended to track the GMPPT of the PV module to impose the system to operate at a specific unique GMPP, where the system can achieve its maximum power. In addition, power electronic circuits are applied with control circuits to attain GMPP operation^14,15^.

**Figure 1:**
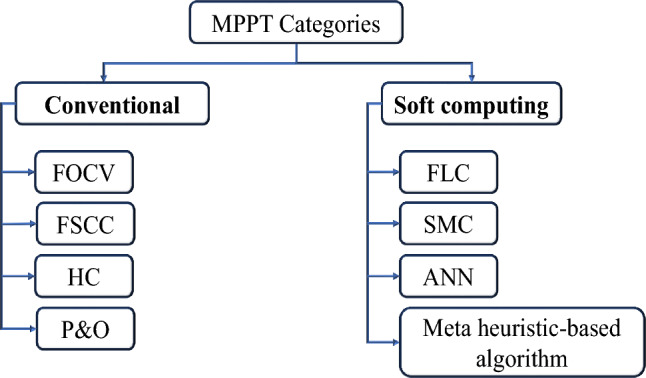
MPPT categories.

Many tracking approaches have been, recently, introduced to attain the optimal power of the PV system, which has drawn considerable interest from researchers^16,17^. Two MPPT categories are broadly classified; conventional and soft computing MPPT algorithms as depicted in Fig. 1. Owing to their low cost and ease of installation, numerous applications typically use the conventional MPPT algorithms, including; fractional open-circuit voltage (FOCV), fractional short-circuit current (FSCC), Hill climbing (HC) and perturb and observe (P&O). These techniques’ primary shortcomings include their poor tracking speed and significant steady-state oscillations, which lower system efficiency^18–20^. To overcome the previous common shortcomings, many computation-based MPPT algorithms have been suggested such as; fuzzy logic control (FLC), sliding mode control (SMC), artificial neural network (ANN), and meta heuristic-based algorithm. However, the main drawbacks of former algorithms, compared to conventional ones, are the long computation-burden and control-system-complexity^21,22^. To lessen the former shortcomings and the impact of partial shade, a number of static and dynamic reconfiguration approaches and control algorithms were suggested^23,24^. The dynamic reconfiguration technique (DRT) calls for a large number of hardware structures, a supervising reconfiguration algorithm to determine the ideal configuration, sensors, and switch matrices to connect PV modules, which increases control complexity and decreases system reliability^25^. In contrast to DRT, static reconfiguration technique (SRT) does not dynamically change the electrical interconnections^26–28^.

Following the completion of the connection, which remains unchanged, the placements of the PV arrays are selected using a certain puzzle. Moreover, switches and other auxiliary circuits are not required while using SRT, which lowers the cost and complexity of installation^29^. In general, there are some essential drawbacks to SRT that must be considered. Long connectivity wires and a considerable number of steps are needed to complete the final reconfiguration matrix. In ref.^30,31^, the Su Do Ku approach has a wide variety of physical PV array reconfigurations. However, it still has a slight drawback that the first column's value is constant, thus submatrix formation and execution consume long time. Regarding the magic-square proposition in^28^, the mismatch losses are greatly reduced. However, as the array reconfiguration process moves toward its final iterations, this methodology becomes time-consuming and difficult. It also calls for several analyses and assumes a lack of the necessary reconfiguration abilities. Moreover, the reliability of dominance square topology (DS) has been proven for many PSC, but conducting it is more challenging^32^. Also, the competence square method (CS) is rarely used as they require complex implementations of switching and sensors^33–35^. In ref.^36^, a varying step size ANN-based MPPT technique is used to mitigate the effect of PSCs on PV systems. In ref.^37,38^, FLC-based on dynamic safety margin (DSM) as an MPPT method to overwhelmed the restrictions of conventional FLC in shading conditions. The limitations of the last two approaches lie in their reliance on human experience and the need for a high-performance controller. In ref.^39^, A novel optimization algorithm called the “θ-modified krill herd (θ-MKH) method” has been introduced to identify the parameters of the SMC. The primary handicap of this type is the discontinuity of the control signal along the sliding surface due to the presence of the sign function. To achieve duty cycle control, the switching frequency must be sufficiently high, leading to the occurrence of chattering^40^. The genetic algorithm presented in^41^ uses solutions to represent the chosen process routes for remanufacturing jobs. To assess the quality of a solution, Monte Carlo simulation is employed, generating a production schedule based on the specified process routes. However, it is difficult to implement and slow computation capability. In ref.^42,43^, A new MPPT technique, utilizing the Particle Swarm Optimization (PSO) algorithm, is introduced. This technique instantly evaluates the duty cycle, eliminating the necessity for PI control loops. It effectively addresses the limitations of conventional direct control methods, especially under partial shading conditions. However, the approach does have a drawback in that it requires significant time delays for the particles to gather towards the MPP, resulting in longer computation times^44^. References^45,46^ propose a MPPT technique which uses Gray Wolf Optimization (GWO). Drawing inspiration from grey wolves, this approach mimics the leadership hierarchy and hunting procedure observed in wildlife. It demonstrates remarkable accuracy in finding high-quality solutions. However, it suffers from its complexity and difficulty of implementation.

A global flexible power point tracking (GFPPT) approach is introduced, which includes the conventional search-skip-judge global MPP method and a vital strategic scheme^47^. Additionally, an adaptive P&O algorithm with enhanced skipping features is employed^48^. To mitigate the drawbacks of previous methodologies, the utilization of analog-to-digital converter (ADC) samples, shared between the MPPT algorithm and the PI controller in^49^. However, challenges associated with being stuck in a local maximum power point (LMPP) within partial shading conditions (PSC) are often encountered by these approaches, which make use of P&O and INC methods. Furthermore, over-scanning behavior is exhibited, with more than 70% of the parameter space being explored by many of them before finally converging to the Global Maximum Power Point (GMPP)^50^.

To mitigate the previous MPPT controller’s limitations, a direct duty-cycle control (DDCC) strategy is proposed. The DDCC technique boosts the overall system efficiency by the steady-state oscillations elimination, hardware simplification, and ease of implementation. Additionally, the DDCC has a fast-tracking speed for GMPPT extraction during PSCs^51^.

Motivated by the former literature survey, this paper’s contributions can be concluded as follows:

- The solar PV tracking efficiency is investigated for using multiple and a single MPPT DC-DC converter under various PSC patterns with constant and realistic radiation data.
- Implementation of a DDCC for MPPT and extraction the GMPP
- Proposing a novel irradiance estimation strategy to reduce the required sensors number.

The residue of the paper is presented as follows; Section “Proposed system modelling” demonstrates the mathematical modeling of the system. Section “Partial shading causes and effects” presents partial shading causes and effects. The proposed methodology of the article is given in Sect. “Proposed methodology”. While Sect. “Efficiency and power compare” describes efficiency calculation and power compare. The proposed system results and discussion are represented in Sect. “Comparison with counterpart approaches”. Finally, Sect. “Proposed system results and discussion” gives a conclusion of the paper and summarizes the article’s outcomes.

## Proposed system modelling

In this paper, two systems are proposed. The first one consists of a PV station connected to a single DC-DC converter. While in another system, each string is connected to a separated DC-DC converter.

### PV cell engineering model

**Figure 2:**
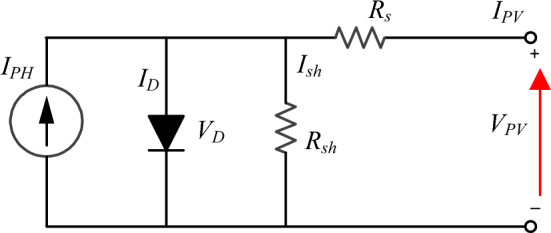
SDM of PV solar cell.

The main components of PV generation systems that convert sunlight to electrical energy for end users are photovoltaic cells. These cells are electrically connected in cascaded and shunt to achieve the desired voltage and current at STC^52,53^. Circuitry modelling of solar cells enables the seamless integration of larger PV systems, including power converters, grid connectivity, etc., using computer aided software^54^. Despite the numerous presented solar cell models, single-diode model (SDM) is widely used owing to its simplicity^55^. Figure 2 presents the single-diode model equivalent circuit^56,57^.

The correlation between voltage and current in solar PV is explained as follows:

1 $$I={N}_{p}{I}_{ph}-{N}_{p}{I}_{sat}\left[{e}^{\frac{q\left(\frac{{v}_{pv}}{{N}_{s}}+\frac{{R}_{s}\cdot {i}_{pv}}{{N}_{p}}\right)}{A\cdot k\cdot T}}-1\right]-\frac{{N}_{p}\cdot \frac{{v}_{pv}}{{N}_{s}}+{R}_{s}\cdot {i}_{pv}}{{R}_{sh}},$$

where *i*_*pv*_ is the solar PV-array generated-current (A), *v*_*pv*_ is the solar PV array terminal voltage (V), *N*_*s*_*—N*_*p*_ are number of cascaded and shunt modules, *I*_*ph*_ is the PV-cell light-generated current (A), *I*_*sat*_ is the reverse saturation current (A), *R*_*s*_—*R*_*sh*_ are series and shunt resistances, respectively, *K* is Boltzmann constant (1.38×10^–23^ J/K), *q* is the electron charge (1.6022 × 10^–19^ C), *A* Is the p–n junction ideality factor, *T* Is the PV cell surface temperature.

The current caused by a photovoltaic module is influenced by several factors, including solar radiation, ambient temperature, cell temperature, and temperature-dependent current coefficient. This formula can be mathematically represented as follows:

2 $${I}_{ph}=\frac{G}{{G}_{0}}\left({I}_{sc}+{K}_{i}\left(T-{T}_{i}\right)\right),$$

where *I*_*sc*_ is the actual short-circuit current (SCC) (A), *G* is the ambient solar emission (W/m^2^), ${G}_{0}$ is the reference solar emission (1000 W/m^2^), *T*_*i*_ is the reference temperature (298 K), *K*_*i*_ is the temperature coefficient of SCC, *T* is the ambient temperature.

The ambient temperature can significantly affect the effectiveness of the solar PV system. Hence, accurate modeling of the temperature dependence of the solar cells is imperative for the optimal design and implementation of the PV system. The reverse-saturation-current of PV-cell is affected by the surrounding temperature, as shown in the following equation:

3 $${I}_{sat}=\frac{{I}_{sc}+{K}_{i}(T-{T}_{i})}{\mathrm{exp}\left(\frac{{V}_{oc}+{K}_{v}\left(T-{T}_{i}\right)}{{V}_{th}}\right)-1},$$

where *V*_*o*_ is the actual open circuit voltage (OCV) of solar PV cell (V), *K*_*v*_ is the OCV coefficient, *V*_*th*_ is the thermal voltage (${V}_{th}=K.\frac{T}{q})$.

Additionally, precise measurement and modelling of the (SCC) are essential for correctly forecasting how well the photovoltaic system would operate under various environmental conditions. The SCC is defined in terms of temperature and irradiation (*T, G*) as follows;

4 $${\mathrm{I}}_{\mathrm{sc}}=\frac{\mathrm{G}}{1000}\left[{\mathrm{ I}}_{\mathrm{sc}\left(\mathrm{STC}\right)}+{\upmu }_{{\mathrm{ I}}_{\mathrm{sc}}}\left(T-{\mathrm{ T}}_{\left(\mathrm{STC}\right)}\right)\right].$$

Also, the diode current of the SDM can be expressed as;

5 $${I}_{D}={I}_{sat}\cdot \left[{e}^{\left(\frac{{V}_{D}}{{N}_{s}\cdot {V}_{th}}\right)}-1\right],$$

where, *V*_*D*_ is diode voltage terminals.

### Open-circuit voltage estimation

This section analyses the anticipated OCV estimation approach considering the impact of temperature and irradiance on the MPP voltage. Hence, an appropriate mathematical formulation is required.

#### Temperature effects

The OCV of PV modules can be formulated as a function of the array’s temperature^58^, as follows;

6 $${V}_{oc}={ V}_{oc\left(STC\right)}+{\mu }_{{V}_{oc}}\left(T-{ T}_{\left(STC\right)}\right),$$

where *V*_*oc(STC)*_ is the OCV at STC, *T*_*(STC)*_ is the temperature of the PV module at STC (*25°C*), *µ*_*Vov*_ is the thermal-coefficient for OCV for PV cell (*V/*^*o*^*C*).

According to (6), the OCV can be determined concerning the solar cell temperature, as develops:

7 $${V}_{oc}={ V}_{oc\left(STC\right)}+{\mu }_{{V}_{oc}}\left(T-{ T}_{\left(STC\right)}\right)\cdot { V}_{oc\left(STC\right)},$$

where ${\mu }_{{V}_{oc}}$ is the OCV thermal-coefficient.

#### Irradiance effects

The effect of irradiance on the OCV can be determined as follows;

In the open-circuit test, *V*_*D*_ and *I*_*D*_ are counterpart to *V*_*oc*_ and *I*_*ph*_, respectively. Consequently, the photo-produced current conveyed in (5) can be symbolized as develops;

8 $${I}_{ph}={I}_{sat}\cdot \left[{e}^{\left(\frac{{V}_{oc}}{{N}_{s}\cdot {V}_{th}}\right)}-1\right].$$

For simplicity, the OCV can be determined as results;

9 $${V}_{oc}={N}_{s}\cdot {V}_{th}\cdot \left[ln\left(\frac{{I}_{ph}+{I}_{sat}}{{I}_{sat}}\right)\right].$$

As, *I*_*ph*_ >  > *I*_*sat*_, then the OCV can be expressed as develops;

10 $${V}_{oc}={N}_{s}\cdot {V}_{th}\cdot \left[ln\left(\frac{{I}_{ph}}{{I}_{sat}}\right)\right].$$

The OCV at STC may be determined as arises;

11 $${V}_{oc(STC)}={N}_{s}\cdot {V}_{th}\cdot \left[ln\left(\frac{{I}_{ph\left(STC\right)}}{{I}_{sat\left(STC\right)}}\right)\right],$$

where *I*_*sat*_ = *I*_*sat(STC)*_.

According to Eqs. (10, 11);

12 $${V}_{oc}-{V}_{oc\left(STC\right)}={N}_{s}\cdot {V}_{th}\cdot \left[ln\left(\frac{{I}_{ph}}{{I}_{sat}}\right)-ln\left(\frac{{I}_{ph\left(STC\right)}}{{I}_{sat\left(STC\right)}}\right)\right].$$

Thus,

13 $${V}_{oc}={{V}_{oc(STC)}+N}_{s}\cdot {V}_{th}\cdot \left[ln\left(\frac{{I}_{ph}}{{I}_{ph\left(STC\right)}}\right)\right].$$

By recognizing the effect of *I*_*ph*_ from (13) into (7);

14 $${V}_{oc}={{V}_{oc(STC)}+N}_{s}\cdot {V}_{th}\cdot \left[ln\left(\frac{{I}_{ph}}{{I}_{ph\left(STC\right)}}\right)\right]+{k}_{th}\left(T-{ T}_{\left(STC\right)}\right)\cdot { V}_{oc\left(STC\right)}.$$

Also, the PV current is directly proportional to the radiation intensity (*G*). Therefore, the OCV of PV modules can be formulated as a function of PV-array’s radiation. Hence, Eq. (13) can be expressed, based on system irradiation, as follows;

15 $${V}_{oc}={{V}_{oc(STC)}+N}_{s}\cdot {V}_{th}\left[ln\left(\frac{G}{{G}_{0}}\right)\right]$$

while ${\mathrm{G}}_{0}$ is the irradiance level at STC (1000 W/m^2^).

### MPP voltage estimation

The MPP-voltage estimation forms a critical task in optimizing the performance of PV-systems, as it affects the MPP efficient extraction from the PV array.

**Figure 3:**
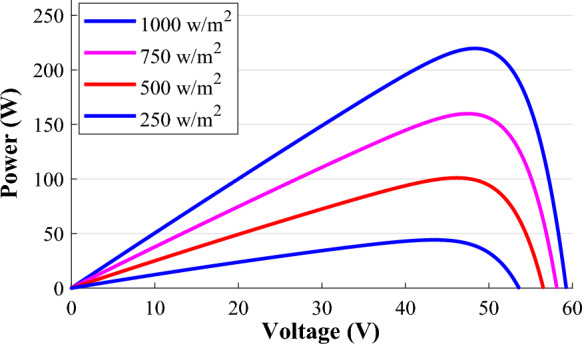
P–V characteristics at various radiation level.

According to Fig. 3, the distribution of MPPs spacing resembles that of open-circuit points. Hence, the MPP can be defined as follows^1^;

16 $${v}_{mpp}={v}_{mpp\left(STC\right)}+{N}_{s}{V}_{th}\mathrm{log}\left(\frac{G}{{G}_{0}}\right).$$

Additionally, there is a linear relevance between the temperature and the OCVs^1^. Since the MPP voltages are seen similarly, hence, the MPP voltage can be expressed as follows;

17 $${v}_{mppt}={v}_{mpp\left(STC\right)}+{\upmu }_{{V}_{oc}}.\left(T-{T}_{o}\right),$$

where ${v}_{mppt}$ is the voltage at MPP due to temperature variant.

Considering (16, 17) and for the complete influence of the environmental factors (radiation and temperature) on the values of MPP voltage, the following conclusion can be reached;

18 $${v}_{mppn}={v}_{mpp\left(STC\right)}+{nN}_{s}{V}_{th}\mathrm{log}\left(\frac{G}{{G}_{0}}\right)+{\mu }_{{V}_{oc}}.\left(T-{T}_{o}\right),$$

where ${v}_{mppn}$ is the MPP voltage at any atmospheric condition. Since the PV array's operating temperature is identical to the temperature at STC, the term referring to temperature variation may be ignored, leading to the equation;

19 $${v}_{mppn}={v}_{mpp\left(STC\right)}+{nN}_{s}{V}_{th}\mathit{log}\left(\frac{G}{{G}_{0}}\right),$$

where n is the diode’s ideality factor. For simplicity; Eq. (19) can be expressed as follows;

20 $${v}_{mppn}={v}_{mpp\left(STC\right)}\left(1+A\mathrm{log}\left(\frac{G}{{G}_{0}}\right)\right),$$

where $$A= \frac{{nN}_{s}{V}_{th}}{{v}_{mpp\left(STC\right)}}.$$

### Radiation estimation

In the PV-system design, a reduction in the system's total number of sensors is necessary because the realistic budget cost is a critical factor. Hence, accurate solar-irradiation estimation is essential for MPP-voltage determination to enhance system reliability and reduce the required number of sensors. Many studies have proposed different strategies for solar irradiance estimation by using the PV-system existing voltage/current sensors^1^. In line with international standards such as IEC 60,891, a new technique has been proposed for irradiance estimation, which can be expressed as follows^5^.

21 $${G}_{est.}={G}_{0}\left[\frac{{i}_{sc(STC)}+\Delta i}{{i}_{sc(STC)}+\frac{{k}_{i}}{{k}_{v}}({R}_{s}\Delta i+\Delta v)}\right],$$

where $\Delta i,\Delta v$ are the variation due to climate change in the PV cell current and voltage, respectively.

The variation in current and voltage of the PV system can be represented as follows:

22 $$\Delta i={i}_{pv}-{i}_{mpp},$$

23 $$\Delta v={v}_{pv}-{v}_{mpp},$$

By substituting the value of the estimated irradiance to evaluate the voltage at MPP, Eq. (20) can be stated as follows;

24 $${v}_{mppn}={v}_{mpp\left(STC\right)}\left(1+A\mathit{log}\left(\frac{{G}_{est.}}{{G}_{0}}\right)\right).$$

### DC-DC converter modelling

**Figure 4:**
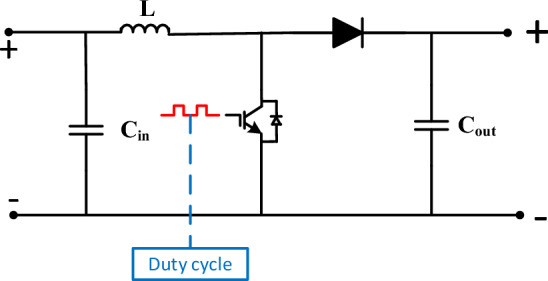
Boost converter model.

DC-DC converters are electronic circuits designed to transform one DC voltage level into another DC voltage level with a specific voltage conversion ratio. A DC-DC boost converter (BC), represented in Fig. 4, controls the PV array’s low and erratic voltage and serves as an mediator between the grid-connected inverter and the PV module circuit^54,59^. With the help of the DC-DC boost converter, one can apply the MPPT controller to confirm the system operation at the MPP^5^. Also, the BC duty-cycle controls the converter's output voltage. Thus, when the switch is opened, the chopper switching state (w) is defined as zero (0), and adjusted to one (1) when the switch is closed^60^, as clearly expressed in the following equation;

25 $$\mathrm{w}=\left\{\begin{array}{c}0 , switch\,\, is \,\,off \\ 1, swith \,\,is\,\, on\end{array}\right.$$

The BC dynamic model can be deduced using voltage and current Kirchhoff’s laws as follows^61^;

26 $$\frac{di}{dt}=\frac{{V}_{PV}-{V}_{out}}{L}+\mathrm{w}\frac{{V}_{out}}{L},$$

27 $$\frac{{dV}_{out}}{dt}=\frac{{i}_{L}}{{C}_{out}}\left(1-\mathrm{ w}\right),$$

where ${V}_{out}$ is the BC ‘s terminal-voltage, ${i}_{L}$ is the inverter input current.

### Direct duty-cycle control

The *DDCC* is employed in PV systems to regulate the produced power of the system by adapting the duty-cycle of the DC-DC converter. It forms a simple and efficient way to handle the power output from the PV systems, which is often used in small-scale and low-cost PV systems^62^. In DDCC, the duty-cycle of the DC-DC converter is adjusted based on the difference between the required and actual output-powers of the PV system. By adjusting the duty cycle, the DC-DC converter can regulate the output voltage and current to accord the required output-power^63^. Using DDCC can help improving the system overall efficiency, which can result in greater energy production and reduced costs over time^64^. The BC voltage transfer-ratio can be expressed in terms of the duty-cycle (*D*), as follows;

28 $$\frac{{V}_{DC}}{{V}_{PV}}=\frac{1}{1-D},$$

where ${V}_{DC}$ is the DC-link voltage at the front end of the utility side converter (GSC). Hence, the duty-cycle of the BC can be obtained directly as follows;

29 $$D=1-\frac{{v}_{mppn}}{{V}_{DC}}.$$

### Grid-side converter modelling

The GSC function is to regulate and control the flow of power between the PV generation system and utility grid. The grid-side converter works by transforming the DC-power produced by the PV generation system into AC-power that can be supplied into the utility grid. In addition, it ensures that the PV-system output is synchronized with the utility frequency and voltage to allow seamless integration. Moreover, the GSC regulates the power factor (PF) of the grid-integrated PV-system for unity PF operation of the proposed system. By maintaining an improved PF, the grid-side converter ensures that the PV system runs at optimal productivity and reduces the risk of system instability and voltage fluctuations^49,65^.

**Figure 5:**
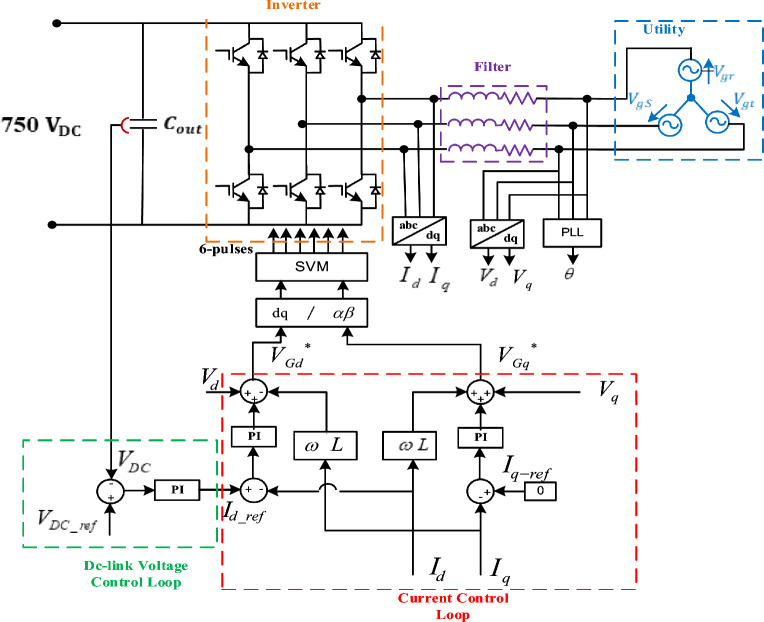
DC-AC converter control scheme.

To control the DC-link voltage and maintain its functional status, the GSC employs a PI-based outer control loop, while the dual PI-based inner control loops are used to adjust the d-q axis currents to its reference ones. A control block diagram illustrating this process is presented in Fig. 5.

Assuming a balanced grid-integrated system, hence, the voltages of three-phase grid can be expressed as develops;

30 $$\left[\begin{array}{c}{e}_{r}\\ {e}_{s}\\ {e}_{t}\end{array}\right]={R}_{f}*\left[\begin{array}{c}{i}_{r}\\ {i}_{s}\\ {i}_{t}\end{array}\right]+{L}_{f}*\frac{d}{dt}\left[\begin{array}{c}{i}_{r}\\ {i}_{s}\\ {i}_{t}\end{array}\right]+\left[\begin{array}{c}{V}_{gr}\\ {V}_{gs}\\ {V}_{gt}\end{array}\right],$$

where ${e}_{r}$, ${e}_{s}$ and ${e}_{t}$ are the GSC line voltages, ${i}_{r}$, ${i}_{s}$ and ${i}_{t}$ are the GSC line currents, ${V}_{gr}$*,* ${V}_{gs}$ and ${V}_{gt}$ are the GSC phase voltages, ${R}_{f}$ is the filter resistance, ${L}_{f}$ is the filter inductance.

The GSC d-q axis voltages or the grid voltages’ rotating reference-frame can be stated as follows;

31 $$\left[\begin{array}{c}{v}_{gd}\\ {v}_{gq}\end{array}\right]=\left[\begin{array}{c}{e}_{d}\\ {e}_{q}\end{array}\right]-{R}_{f}\cdot \left[\begin{array}{c}{i}_{d}\\ {i}_{q}\end{array}\right]-\left[\begin{array}{c}\dot{{\lambda }_{d}}-{\omega }_{0}{\psi }_{q}\\ \dot{{\lambda }_{q}}+{\omega }_{0}{\psi }_{d}\end{array}\right],$$

where *v*_*gd*_, *v*_*gq*_, *e*_*d*_, and *e*_*q*_ are the inverter d and q axes voltage components of the utility voltages, respectively, ${\omega }_{0}$ is the angular frequency of the grid (rad/s).

The GSC exchanges no reactive power with the utility to achieve the unity PF. The two-power component can be expressed instantaneously in the d-q axis representation as follows;

32 $$P=\frac{3}{2}{(V}_{gd}{i}_{d-}{V}_{gq}{i}_{q}),$$

33 $$Q=\frac{3}{2}{(V}_{gd}{i}_{q-}{V}_{gq}{i}_{d}),$$

## Partial shading causes and effects

**Figure 6:**
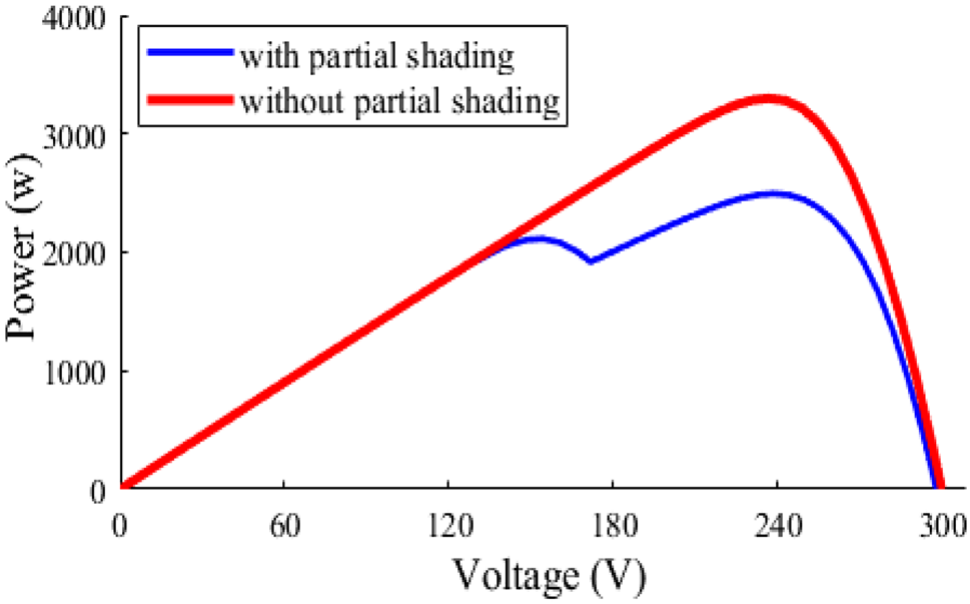
P–V characteristic curve under PSC.

When one or more panels in a series string are shaded, the output-current of the whole string decreases, which constraints the current flow in the entire string and reduces the overall output-power of the PV-system^66,67^. Utilizing bypass diodes, which enable current to pass through the unshaded cells in the string, is one method of addressing PS. Bypass diodes are connected in shunt with each solar cell or group of them, and are designed to activate when the voltage across the cells drops below a certain threshold. By redirecting the current around the shaded cells, bypass diodes reduce the harmful effects of PS on the performance of the PV system. This enables the rest of the module to operate at higher efficiency and avoid the PV-system overheating^8,9^. This article studies the performance of the PV-system under various PSCs. Figure 6 depicts PV module’s characteristic curve, which exhibits a unique MPP as it exposed to a uniform irradiance^68^.

However, when the module is subjected to different levels of radiation, it exhibits multiple LMPPs based on the shading patterns. Among these LMPPs, there is a unique GMPP that offer optimal operating power of the shaded system^69–71^. Also, understanding the behaviour of PV modules under PS is quite important to enhance the system performance and operating efficiency. There are two types of partial shading that are possible to exists on the PV array: static shading, and dynamic shading. The static shading refers to a specific shadow that remains on the PV array for a period of time, where the dynamic shading is the varying shades on the surface of PV array that results from a moving cloud or swaying tree branches caused by the wind^19,72–74^.

## Proposed methodology

In this study proposes a reconfiguration of the PV-system to suppress the negative impact of PS on the PV-system performance by dividing the PV-system into multiple parallel strings. In this work, the proposed system comprises four strings, with each string comprises three paralleled sub-strings. Each sub-string involves of five cascaded-connected modules. The PV-station studied in this work consists of 60 Canadian CS5P-220 M panels, with a total power of 13.2 kW. Table 1 lists the PV-module specifications at STC.

**Table 1:** Canadian CS5P-220 M PV unit specifications.

Optimal power, *P*_*MPP*_	220 (Watt)
Voltage at MPP, *V*_*MPP*_	48.3159 (Volt)
Current at MPP, *I*_*MPP*_	4.54758 (Ampere)
OCV, *V*_*OC*_	59.2618 (Volt)
SCC, *I*_*SC*_	5.09261 (Ampere)
Ambient temperature, at STC	25 (Celsius degree)

**Figure 7:**
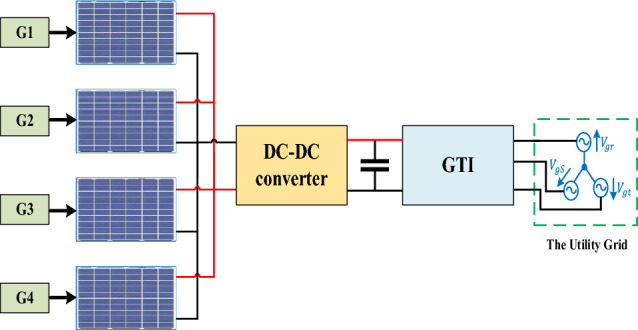
Single-MPPT system configuration.

Figure 7 depicts the PV system’s single-MPPT (SMPPT) arrangement, in which a single DC-DC converter is connected to four parallel strings of solar panels. The usage of an SMPPT in PV systems has a number of drawbacks that may reduce the system's overall performance and efficiency such as;

- First off, an SMPPT can only track the MPP of a single PV module or string at a time. Hence, the system will only function at the MPP of the weakest module or string if numerous modules or strings are linked in parallel that diminish the system efficiency.
- Second, the usage of a SMPPT makes the PV-system vulnerable to shading, as even a small shading area on single module/string causes the entire system to operate at a sub-optimal MPP, leading to a reduction in system output power.
- Third, the configuration of SMPPT encounters difficulties in locating the system's global MPP. Consequently, there is a need to identify a controller capable of rectifying this issue, which increases the system complexity and the required controller’s specifications. For instance, if a cloud passes over the PV system, the output voltage and current of each module or string may change, and a SMPPT may not be able to adjust to these changes quickly enough, leading to reduced power output^22,25,75,76^.

**Figure 8:**
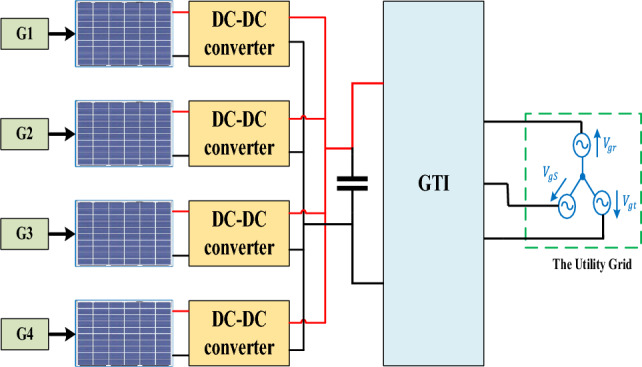
Multi-MPPT system configuration.

To overcome the limitations of SMPPT configuration, Multi-MPPT (MMPPT) approach offers optimal MPPT under different levels of shading conditions, as shown in Fig. 8. Each string is tied to a DC-DC converter, which enables optimal power extraction of each individual string of PV solar panels.

Hence, results in increased energy conversion efficiency. In comparison to SMPPT, MMPPT offers more dependability by enabling sustained operation even in the case of a problem with one of the PV solar panel strings. Additionally, it provides more design flexibility for PV systems because it can support a variety of solar panel counts and configurations and is simple to adapt to changing power needs. Additionally, by maximizing the power output of each string of solar panels, MMPPT arrangement lowers the cost of the PV solar system and enables the use of smaller, more affordable power converters^32,77^. Table 2 lists the parameters of the DC-DC converters.

**Table 2:** Parameters of the DC-DC converters.

Single-MPPT	L1	10 (mH)
	C1	14 ($\mu F$)
	FSW	5000
Multi-MPPT	L2	3 (mH)
	C2	14 ($\mu F$)
	FSW	20,000

**Figure 9:**
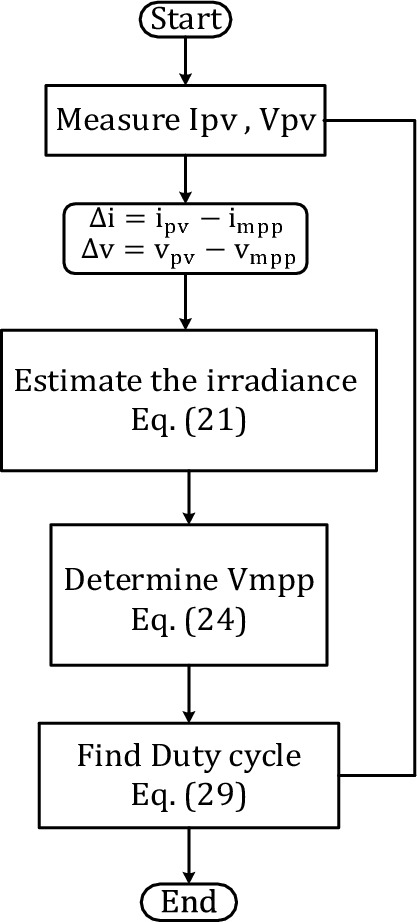
Proposed control strategy.

Figure 9 depicts the flow chart of the projected control approach. The first step is to measure the voltage and current of PV module, then calculate the difference between these values and voltage and current of MPP at any shading condition. We use this subtraction product to estimate the radiation. The estimated radiation is used to evaluate the estimated voltage, hence, the duty cycle of the converter.

## Efficiency and power compare

This section provides comparison between SMPPT and MMPPT configurations under varying irradiance profiles. The primary factors considered in this comparison are the average output power and voltage ripples. The tracking efficiency of each system can be readily determined through the following formula:

34 $$\eta =\frac{{P}_{act.}}{{P}_{th.}}.100 \left[\%\right],$$

where ${P}_{act.}$ and ${P}_{th.}$ are the actual and theoretical PV output power, respectively^78^.

## Comparison with counterpart approaches

This section compares the proposed MPPT method with some recent approaches. The comparative analysis is established considering the system capability to track the GMPP under the partial shading condition, the system tracking response, system efficiency, and the contained steady-state oscillations (SSO). Evidently, Table 3 presents a comparative analysis between the proposed MPPT method and recent approaches. This analysis highlights the efficiency of the proposed method in accurately tracking the GMPP during Partial Shading Conditions. Furthermore, it demonstrates the fast tracking speed of the proposed methodology. In addition, the proposed strategy diminishes the system high-frequency steady-state oscillations, which enhances the control system design and its reliability for practical use. Despite the parallel operation of Multi-MPPT that may enquires many calculations, the proposed system shows a simple MPPT control system compared to most of the recent topologies, which lowers the required specifications of the controller for real system implementation.

**Table 3:** Comparison with other literature.

Name of method	Tracking of GMPP	Tracking speed	Efficiency	SSO
^79^	Yes	Slow	Sufficient	Yes
^80^	No	Moderately	Low	Yes
^81^	No	Good	Low	Yes
^27^	No	Not reported	High	Yes
^13^	No	Fast	High	Yes
Conventional P&O	Yes	Fast	High	Yes
proposed method	Yes	Fast	High	No

## Proposed system results and discussion

**Figure 10:**
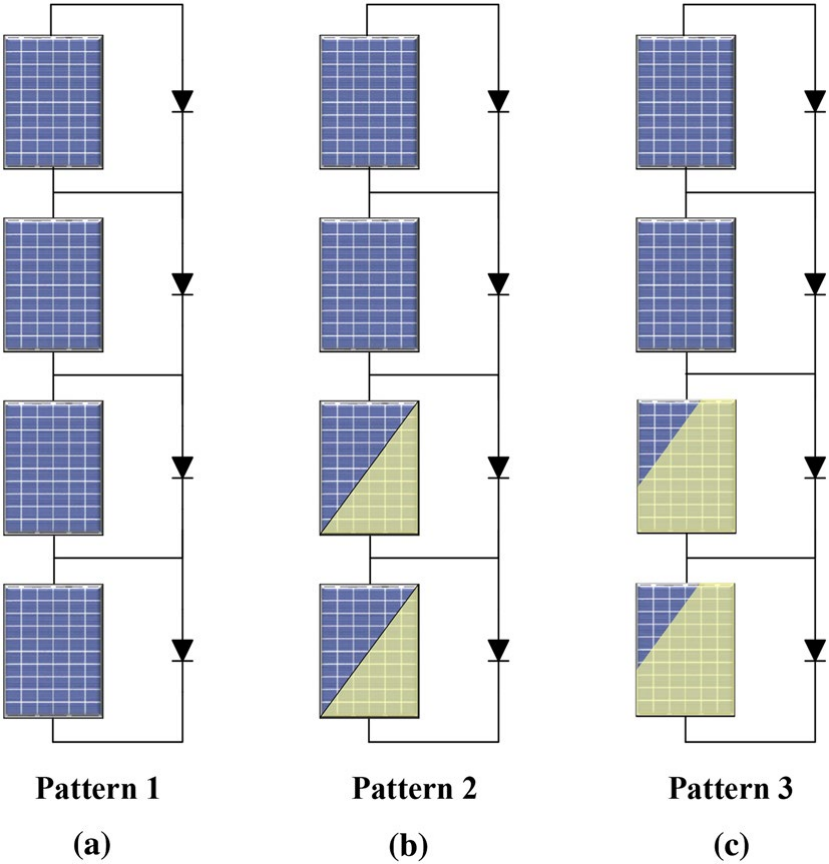
Studied shading pattern on the proposed system.

As previously illustrated, the dual-stage grid-connected PV-system has been studied considering two configurations. The two configurations are constructed using MATLAB/Simulink computer-aided software. In addition, three solar irradiance patterns, see Fig. 10, are applied to the former PV-system configurations, i.e. SMPPT and MMPPT, for a fair comparison between the selected configurations. The purpose of this study is to know which configuration has reliable functioning under various PSCs. The following results show the estimated solar radiation, PV output-voltage, PV output-power and the converter duty-cycle of the converter for MPPT.

### Shading pattern-1

In shading pattern-1, all the PV panels are subjected to a uniform irradiance of 1000 W/m^2^, which is considered as the STC. Under these conditions, the optimal output power at STC is 13.2 kW. First of all, the proposed dual-stage system is tested under STC conditions to illustrate the system performance characteristics and to ensure the control system robustness. Also, by conducting the STC tests, the results obtained can be used as a benchmark to evaluate the system’s performance under other operating conditions. Therefore, these findings are vital for the design and optimization of PV-systems, which provide valuable insights into the system’s efficiency and performance under ideal conditions.

**Figure 11:**
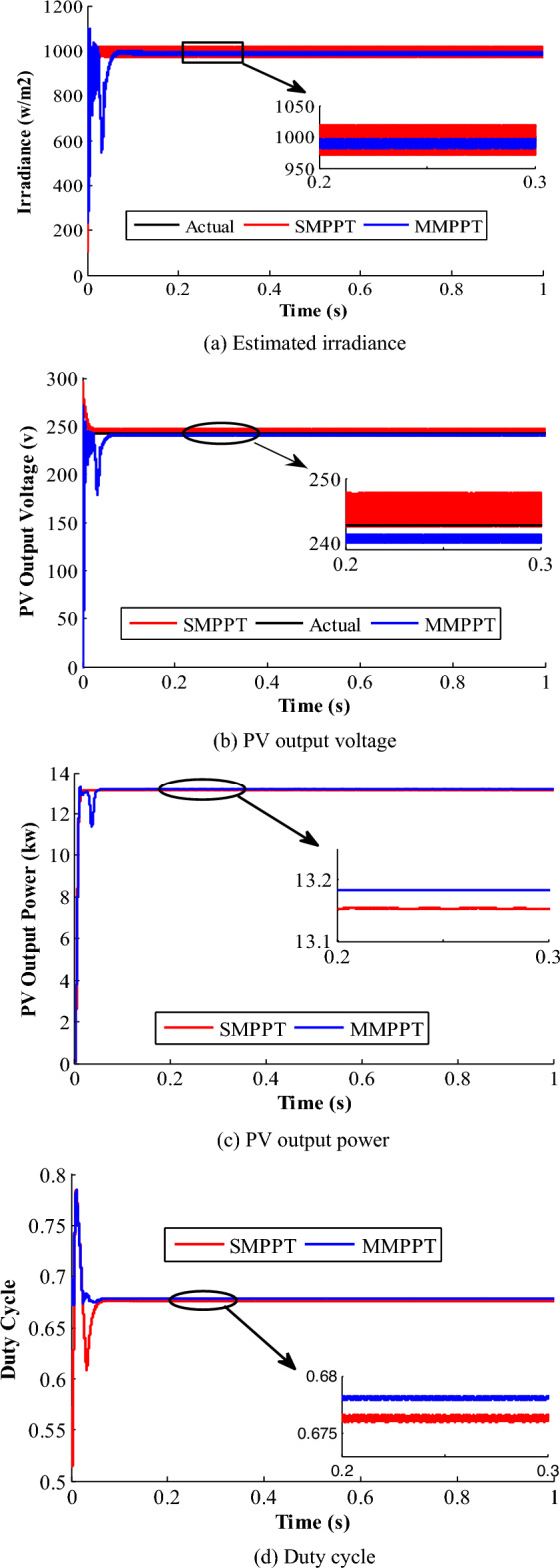
Results of pattern 1.

Figure 11 shows the PV-side results of the proposed grid-tied system considering both SMPPT as well as MMPPT configurations. As shown in Fig. 11a, the SMPPT-based estimated radiation profile exhibits steady-state oscillations between 970 W/m^2^ and 1020 W/m^2^, while displays steady-state oscillations between 985 W/m^2^ and 998 W/m^2^ for the MMPPT PV-system configuration. Obviously, it confirms the accurate operation of the MMPPT-based DDC control-loop with low peak-to-peak (PTP) steady-state oscillations compared to SMPPT-structure. The irradiance is estimated in order to decrease the number of sensors used in the system installation, hence diminishing the cost of operation. Besides, the PV output voltages of the intended system, according to system specifications listed in Table 1, are depicted in Fig. 11b. According to the characteristics of the PV panels in Table 1, the voltage at which optimal power point is extracted at STC is 241.5 V. The SMPPT-based PV-system shows a PTP steady-state voltage oscillations of 5.3 V, where the system exhibits only 0.5 PTP steady-state voltage for the MMPPT system configuration. Hence, the reduced oscillations level of the proposed PV-system enhances the system power quality, elements voltage and current stresses, power loss, and improve its overall efficiency. Figure 11c shows the PV generated power of the two configurations. The average generated power of SMPPT is 13.15 kW, with an efficiency of 99.7%, while MMPPT’s average power is 13.18, with efficiency of 99.85%.

The shift from the optimal voltage and the oscillation levels plays a crucial role in contributing to difference in the output power values. Also, the duty-cycles are illustrated in Fig. 11d. The settling time and the oscillation level in MMPPT are smaller than that of SMPPT.

### Shading pattern-2

**Figure12:**
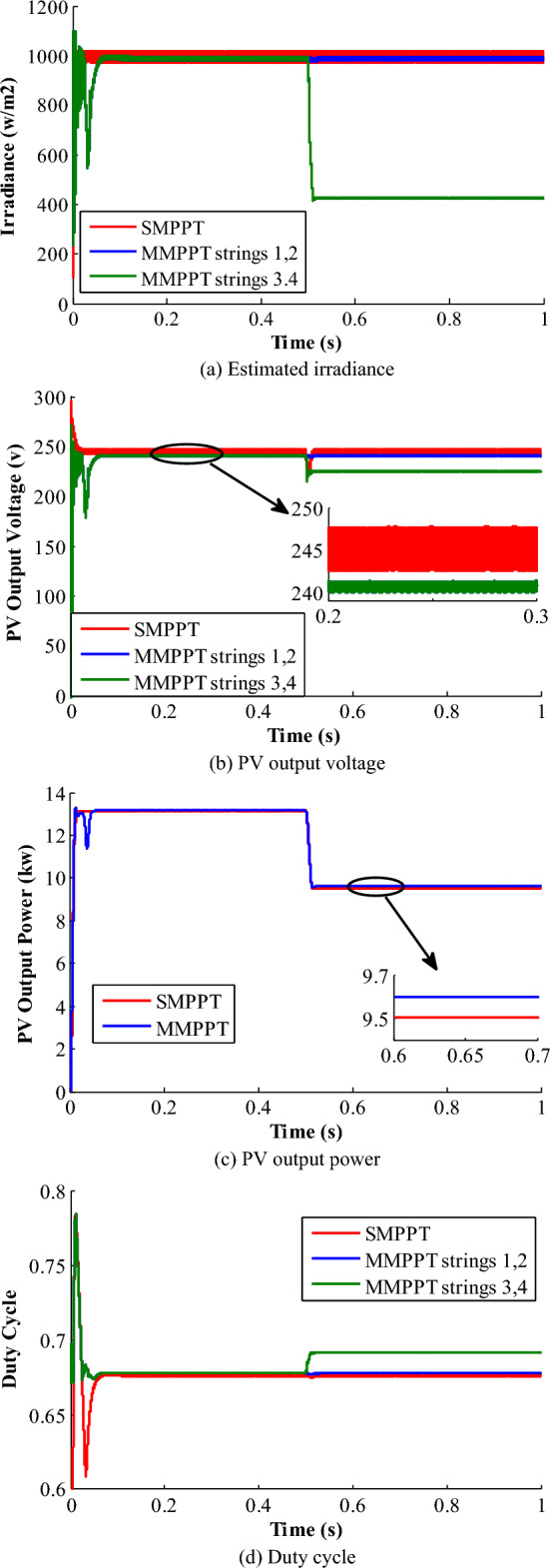
Results of pattern 2.

In this scenario, the PV-system is subdivided into two levels of irradiations: the first level forms a constant irradiation profile of 1000 W/m^2^ for first two strings (1st & 2nd strings). Second irradiance level is exposed to the remaining two strings (3rd & 4th strings), in which a uniform irradiance profile of 1000 W/m^2^ is applied for 0.5 s and then stepped down to 500 W/m^2^ at t = 0.5 s for another 0.5 s, as depicted in Fig. 12.

This pattern protests the performance of the two configurations and the anticipated control strategy. Figure 12a shows the estimated irradiance profiles of the SMPPT and MMPPT configurations. The SMPPT-based estimated irradiance does not correctly track the actual radiation profile, as the irradiance sensor is installed at the first string, which is always exposed to 1000 W/m^2^. However, the MMPPT-based estimated irradiance profile performs as an online tracking strategy for irradiance level variation at any location of the PV-system. Moreover, the effect of irradiance variation on the PV-system performance, power quality, and efficiency is less compared to the SMPPT configuration. Figure 12b shows the PV-system estimated-voltage for both configurations. In the SMPPT configuration, the PV-system estimated-voltage remains unchanged following the same behaviour of the wrong estimated radiation. Also, the average value of the estimated-voltage at the MPP is 242.7 V and the oscillation level is around 4 V PTP. In MMPPT configuration, the estimated output-voltage from the first two strings is depicted in Fig. 12b with the blue colour. The average output-voltage is 240.5 V, and the oscillation level is 0.5 V PTP. The estimated output voltage of the last two strings is shown in green colour. The average output voltage is 225.29 V with an oscillation level of 1.3 V, whereas the MPP-voltage is 230.7 V at 500 W/m^2^.

The PV generated-powers of the two PV-system configurations are depicted in Fig. 12c. At the time interval between (0–0.5 s), it shows a performance similar to pattern-1. At the second interval (0.5–1 s), the PV-system output-powers are decreased due to the effect of radiation. The theoretical optimal output-power is 9.62 kW under radiation of 500 w/m^2^. For the SMPPT structure, the average output-power is 9.51 kW with 98.85% system efficiency. In the other side, the average output-power of the MMPPT configuration is 9.6 kW with a tracking efficiency of 99.79%. In addition, the converter duty-cycles for both SMPPT and MMPPT configurations are portrayed in Fig. 12d. Obviously, the duty-cycles of both cases remain unchanged during the first 0.5 s due to the uniform applied irradiance profiles.

However, the duty-cycles of the SMPPT remain unchanged during the time interval (0.5–1 s) despite the irradiance change, which reveals the tracking system weakness under PCSs. On the other side, the duty-cycle of the 3rd and 4th strings of the MMPPT changed for voltage boosting, which is portrayed with green color in Fig. 12d. Moreover, Fig. 12 confirms the effectiveness of the MMPPT for shaded and unshaded PV-system, compared to the SMPPT configuration. These results show that the voltage stability in MMPPT configuration is better than that of SMPPT.

### Shading pattern-3

**Figure 13:**
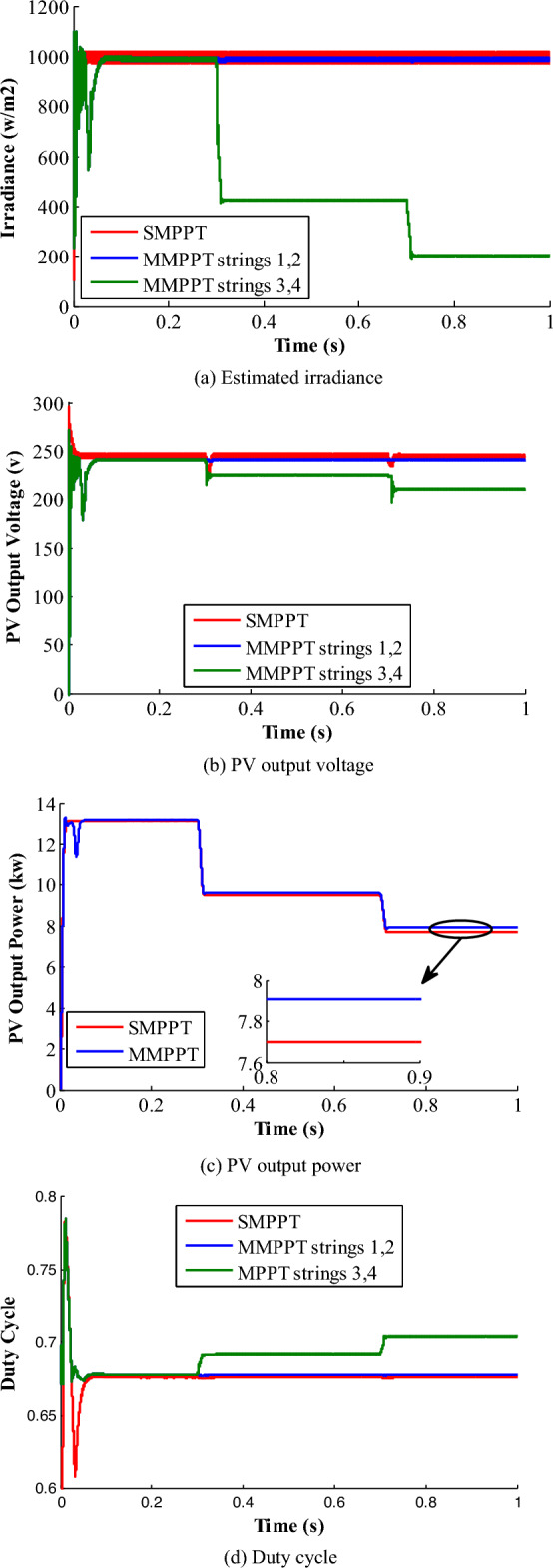
Results of pattern 3.

In this pattern, the radiation on the first two strings is a uniform irradiance profile of 1000 W/m^2^. On the other two strings, the radiation has two step-changes. First, the radiation starts with 1000 W/m^2^ at all strings for 0.3 s (0–0.3 s), changes to 500 W/m^2^ for 0.3 s (0.3–0.6 s) and decreased to 250 w/m^2^ for 0.4 s (0.6–1 s) as represented in Fig. 13. Figure 13a shows the estimated radiation of the SMPPT and MMPPT configurations. In SMPPT configuration, the results remain similar to the mistaken results of the previous pattern-2 as the irradiance sensor is installed at the first string. In the MMPPT, the estimated radiation always tracks the actual radiation as cleared in the green color of Fig. 13a. Similarly, the estimated output-voltage of two configurations is illustrated in Fig. 13b. The average estimated voltage of SMPPT is 242.7 V with an oscillation level of 5.3 V, as depicted with red color. Also, the estimated voltage of the first two strings is depicted in blue color, which has a uniform irradiance level of 1000 w/m^2^. This voltage has an average value of 240.6 V with an oscillation value of 1.3 V PTP. The other voltage tracks the changes in the radiation, exposed to the third and fourth strings, illustrated in green color. For 500 w/m^2^ radiation level, this voltage has an average value of 225.29 V with a PTP oscillation level of 1.3 V. For irradiation level of 250 w/m^2^, the estimated voltage is 210.13 V, while the optimal voltage at MPP is 217.7 V and the PTP oscillation level is 1.3 V. Figure 13c shows the output power of the two PV-system configurations. The optimal power in the case of 250 W/m^2^ is 7.92 kW. Obviously, the power obtained from the MMPPT is higher than the power generated from the SMPPT configuration. In the time interval from 0.7 to 1 s under 250 w/m^2^.

The average output-power in from SMPPT and MMPPT configurations are 7.65 and 7.9, respectively, and the percentage efficiencies of the two configurations are 96.92 and 99.7, respectively. Figure 13d shows the duty-cycle generated by the controller. Duty cycle does not change in case of SMPPT. In MMPPT configuration, the duty-cycle tracks the estimated-irradiance profile and achieves estimated voltages close to the V_MPP_ at different radiation levels. However, it remains unchanged for the SMPPT configuration.

### Comparative analysis between SMPPT and MMPPT configuration

This section provides a comparative view between the two proposed systems. Tables 4 and 5 summarize all results of SMPPT and MMPPT, respectively. The first column shows the pattern number as explained previously. The second column declares the time of each period in the specified pattern. The next two columns illustrate the operating voltage and the duty cycle of each DC-DC converter, respectively. The following column shows the output power of the system under pattern, the last column exhibits the efficiency of the approach under any studied pattern to make a comparison between two approaches.

**Table 4:** Result of single-MPPT configuration.

	Time (s)	Voltage (V)				Duty cycle			Power (kW)			Optimal power (kW)	Efficiency (%)
		Strings 1 & 2	String 3	String 4	${\mathrm{V}}_{\mathrm{mmp}}$	Strings 1 & 2	String 3	String 4	String 1 & 2	String 3	String 4		
Pattern 1	0 to 1	**242.77**			241.5	0.676			13.15			13.2	99.77
Pattern 2	0 to 0.5	**242.77**			241.5	0.676			13.15			13.2	99.77
	0.5 to 1	**242.77**	*242*.77		230.7				9.51			9.62	98.85
Pattern 3	0 to 0.3	**242.77**	**242.77**		241.5	0.676			13.15			13.2	99.77
	0.3 to 0.7	**242.77**	*242.77*		230.7				9.51			9.62	98.85
	0.7 to 1	**242.77**	***242.77***		217.07				7.68			7.924	96.92

Bold—1000 W/m^2^, Italics—500 W/m^2^ and Bold italics—250 W/m^2^.

**Table 5:** Result of multi-MPPT configuration.

	Time (s)	Voltage (V)					Duty cycle				Power (kw)						Efficiency (%)
		String 1	String 2	String 3	String 4	${\mathrm{V}}_{\mathrm{mmp}}$	String 1	String 2	String 3	String 4	String 1	String 2	String 3	String 4	Total power	Optimal power	
Pattern 1	0 to 1	**240.6**	**240.6**	**240.6**	**240.6**	241.5	**0.679**	**0.679**	**0.679**	**0.679**	**3.29**	**3.29**	**3.29**	**3.29**	13.16	13.2	99.85
Pattern 2	0 to 0.5	**240.6**	**240.6**	**240.6**	**240.6**	241.5	**0.679**	**0.679**	**0.679**	**0.679**	**3.29**	**3.29**	**3.29**	**3.29**	13.16	13.2	99.85
	0.5 to 1	**240.6**	**240.6**	*225.29*	*225.29*	230.7	**0.679**	**0.679**	*0.699*	*0.699*	**3.29**	**3.29**	*1.51*	*1.51*	9.6	9.62	99.79
Pattern 3	0 to 0.3	**240.6**	**240.6**	**240.6**	**240.6**	241.5	**0.679**	**0.679**	**0.679**	**0.679**	**3.29**	**3.29**	**3.29**	**3.29**	13.16	13.2	99.85
	0.3 to 0.7	**240.6**	**240.6**	*225.29*	*225.29*	230.7	**0.679**	**0.679**	*0.699*	*0.699*	**3.29**	**3.29**	*1.51*	*1.51*	9.6	9.62	99.79
	0.7 to 1	**240.6**	**240.6**	***210.13***	***210.13***	217.7	**0.679**	**0.679**	***0.719***	***0.719***	**3.29**	**3.29**	***0.66***	***0.66***	7.9	7.924	99.7

As the estimated radiation is provided for the first string, values of estimated voltages and duty cycle do not change with pattern variation. These values have an average value of 242.7 V and 0.676, respectively. The constancy of this value can be attributed to the fact that the SMMPT estimation is exclusively conducted for the first string within the system. In this initial pattern, the voltage closely approximates Vmpp. However, as we move to the subsequent two patterns, the voltage differential between them expands, resulting in decreased system efficiency, as evidenced by the power and efficiency columns. This configuration leads to misalliances and reduction in the generated power. The system with SMPPT configuration cannot withstand under severe weather condition change. By comparing the output power extracted from the system and the optimal power, more shading runs less efficiency. At radiation values of 1000, 500 and 250 W/m^2^, the PV out power is 13.15, 9.51 and 7.68 kW, respectively. Also, the system efficiency for the aforementioned radiation is 99.77, 9.85 and 96.92%, respectively. The system efficiency has distorted with alteration of radiation level, which is not appropriate operation under PSCs.

On the other hand, Table 5 clarifies the results of MMPPT configuration. The MMPPT strategy extracts the MPPs of each string individually under any weather condition. The values of voltages and duty cycle changed with radiation deviation, and be more nearer from the V_MPP_ than that of SMPPT configuration. At radiation levels of 1000, the PV voltage is 240.6 V while the voltage at MPP is 241.5 V. In the second level of radiation, the PV output voltage is 225.29 V while V_MPP_ is 230.7 V, compared this value with the unchanged value of the SMMPPT of 242.7 V. At the lowest radiation, the PV output voltage and V_MPP_ are 210.13 V and 217.7 V, respectively. The PV generated power and efficiency from the MMPPT configuration at the three radiation levels are; 13.16 kW and 99.85%, 9.6 kW and 99.79% and 7.9 kW and 99.7%, respectively.

The efficiency of MMPPT configuration is almost constant over all shading patterns which demonstrates the effectiveness and validation of the proposed methodology. The assessment has been accomplished by observing the performance of the systems under different radiation patterns. The efficiency is computed across all time periods to assess the dynamic performance of the proposed scheme.

### Realistic irradiance data

**Figure 14:**
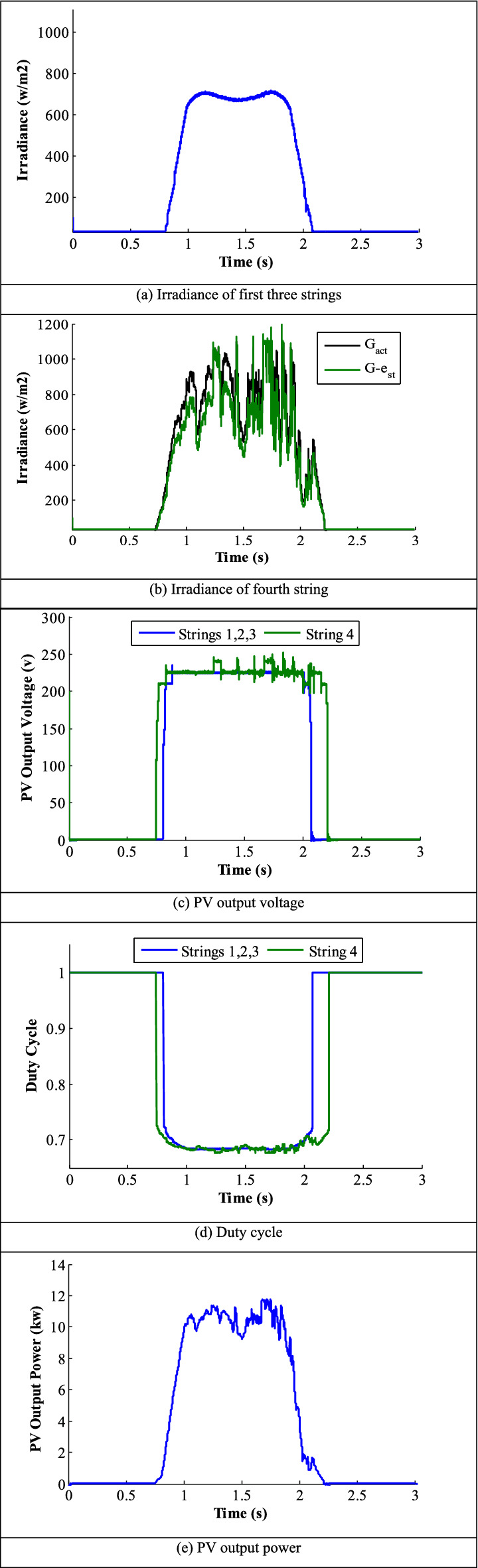

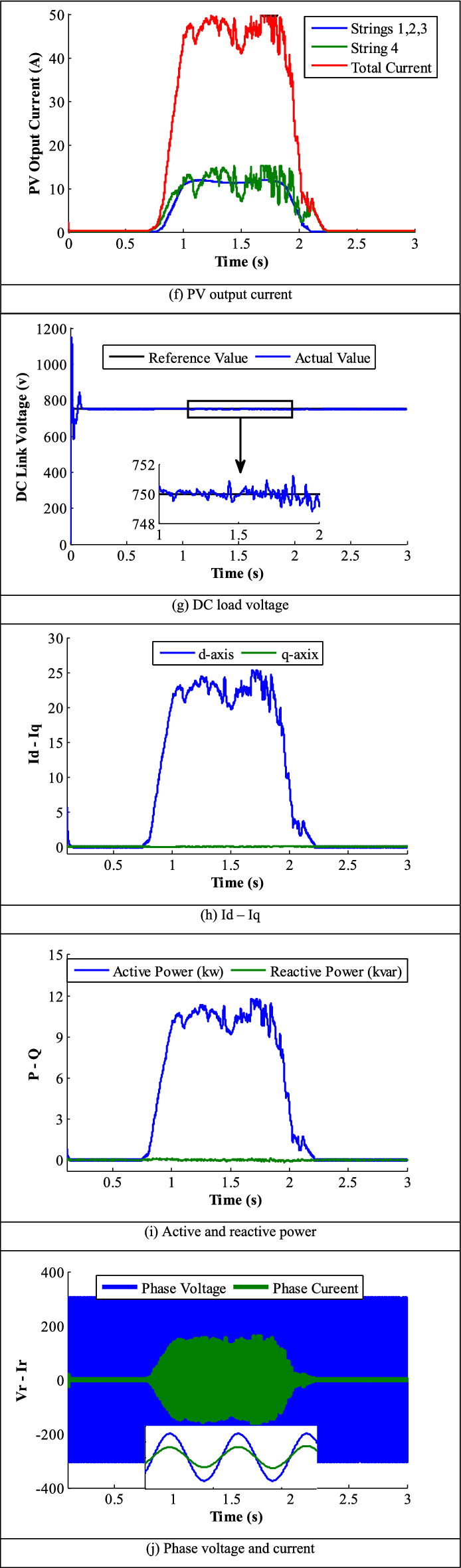
Results of real data.

To ensure the validity of the intended dual-stage PV-system, the following study conducts the system investigation considering a real irradiance profile of Benban city, which located in the south of Egypt^82^, see Fig. 14. Two irradiance profiles are employed in this study; the first profile represents the normal radiation that is applied to the first three strings as detected in Fig. 14a. Figure 14b provides a visual representation of the estimated irradiance for the fourth string. The controller accurately tracks the actual radiation profile and provides the estimated radiation. Figure 14c illustrates the PV-output voltage, which includes fluctuations due to non-uniform irradiance. In addition, the DDCC is used to generate duty-cycle for the converters. Figure 14d shows each duty-cycle waveform that corresponds to its voltage value in Fig. 14c. Obviously, the DDCC successfully adapts the rapid changes in solar radiation profile. Also, the PV output-power from the PV-module, shown in Fig. 14e, closely follows the irradiance curve as expected. At zero radiation, the net output power is almost zero and gradually increases to a maximum value, and then decays again to zero at 2.1 s. In Fig. 14f, the PV output-currents of normal irradiance strings are identical as shown in the blue color, while the current generated by the fourth string is presented in the green color with the same pattern as the irradiance profile. In summary of PV side results, the presented results demonstrate the effectiveness of the controller to accurately track the actual radiation profile even under tough and real irradiance profiles with reduced contained error. Also, the PV output power closely follows the irradiance curve, and the system can adapt to non-uniform irradiance and changing weather conditions over time. Grid side results illustrate the DC-link voltage, grid active and reactive powers, grid d-q axes currents, and the grid-voltages and currents. Figure 14g displays the reference DC-link voltage, fixed to 750 V, and the actual voltage measured at the inverter’s front-end terminals. As seen in Fig. 14g, the system displays a reduced oscillation level, even under rapid irradiance variations. Figure 14h illustrates the d-q axes currents of the system. The d-axis current typically tracks the irradiance profile and power curve, while the q-axis remains at zero that confirms the unity PF operation of the grid-tied system. Also, Fig. 14i depicts the active and reactive power obtained by the system, in which the active power follows the irradiation patterns, and the reactive power is almost zero. Furthermore, Fig. 14j shows the grid phase-voltage and current, which are in-phase that approves the unity PF operation of the system.

The results demonstrate the PV-system transient and steady-state stability, and reliability for grid-integrated applications. The presented findings provide precious insights into the performance and behavior of the dual-stage PV-system considering both SMPPT and MMPPT configurations. These results are of significant importance for selecting PV-system configuration and design of grid-connected systems in the future.

## Conclusion

In this paper, an investigation into the use of a MMPPT configuration as an alternative to SMPPT configuration to address the issue of partial shading in photovoltaic systems. The contributions are twofold: firstly, implementation of radiation estimation technique to reduce system costs by minimizing the required number of sensors. Secondly, using a direct duty cycle method to determine the converters’ duty cycles, simplifying the control system. This paper proposes and simulates two systems using MATLAB-Simulink, comparing the SMPPT and MMPPT systems under varying radiation profiles. Under these conditions, the average efficiency of the SMPPT system is found to be 98.98%, while the MMPPT system achieves an efficiency of 99.81%. These findings validate the proposed approach. A real radiation dataset from Benban, a location in southern Egypt, is used in MMPPT configuration. The results demonstrate that the proposed control system enhances overall system effectiveness while reducing installation costs. These findings hold significant importance as they offer a practical solution to mitigate the challenges of partial shading and enhance the efficiency of photovoltaic systems. The proposed methodology can be readily applied to future PV system designs, offering improved performance and cost savings. Additionally, the approach enables the system to operate at higher efficiency levels. It is worth noting that the proposed control system is characterized by its simplicity, adaptability to rapid climate changes, and robust performance during operation.

## Data Availability

The datasets generated during and/or analyzed during the current study are available from the corresponding author on reasonable request.
